# Outcome for sinonasal malignancies: a population-based survey

**DOI:** 10.1007/s00405-021-07057-0

**Published:** 2021-09-12

**Authors:** Anna Hafström, Johanna Sjövall, Simon S. Persson, Johan S. Nilsson, Christer Svensson, Eva Brun, Lennart Greiff

**Affiliations:** 1 Department of ORL, Head & Neck Surgery, Skåne University Hospital, 221 85 Lund, Sweden; 2 Department of Clinical Sciences, Lund University, Lund, Sweden; 3 Department of Oncology, Skåne University Hospital, Lund, Sweden

**Keywords:** Head and neck cancer, Sinonasal cancer, Mucosal melanoma, Adenocarcinoma, Multimodal cancer therapy, Head and neck surgery

## Abstract

**Purpose:**

Sinonasal malignancies (SNM) represent a rare and complex group of cancers that includes a wide range of histopathological subtypes. Data from population-based cohorts are scarce but warranted as a basis for randomized controlled treatment trials (RCTs). Our aim was to assess overall and histology subset-specific outcomes for SNM patients treated at a tertiary referral centre.

**Methods:**

A retrospective, population-based, consecutive cohort of patients with SNMs diagnosed from 2001 through 2019 was examined. Outcome was analysed in relation to age, gender, site, stage, histopathology, and treatment.

**Results:**

Two-hundred and twenty-six patients were identified, whereof 61% presented with stage IV disease. 80% completed treatment with curative intent, which comprised surgery with neoadjuvant (29%) or adjuvant (37%) radiotherapy, monotherapy with surgery (22%), definitive chemoradiotherapy (7%), or radiotherapy (5%). Median follow-up was 106 months. The 5- and 10-year overall survival rates were 57% and 35%, respectively. Median overall survival was 76 months (esthesioneuroblastoma: 147 months; adenocarcinoma: 117; salivary carcinoma: 88; mucosal melanoma: 69; squamous cell carcinoma: 51, undifferentiated carcinoma: 42; neuroendocrine carcinoma: 9; and NUT-carcinoma 5). The 5- and 10-year disease-free survival rates were 63% and 54%, respectively, and disease-specific survival 83% and 66%. Increasing age, stage IVB, melanoma histopathology, and treatment with definitive chemoradiotherapy emerged as significant independent prognostic risk factors for disease-specific mortality (*p* ≤ 0.001).

**Conclusion:**

The results indicate a seemingly good outcome in comparison to previous reports, particularly for mucosal melanoma, adenocarcinoma, and undifferentiated carcinoma. The study provides additional background for future RCTs focusing on histology subset-specific treatment for SNM.

## Introduction

Sinonasal malignancies (SNM) represent a rare and complex group of cancers that characteristically includes a wide range of histopathological subtypes [1]. The treatment comprises surgery and radiotherapy (RT) with or without chemotherapy (CT) [2], and 5-year overall survival (OS) ranges from 20 to 50% [3]. With few exceptions [4–6], there is limited information on characteristics and fate of SNM derived from population-based cohorts. Arguably, such information is imperative to accurately design future randomized controlled trials (RCTs) and, thereby, to evaluate treatments in relation to specific SNM subsets.

The three Nordic studies by Thorup et al., Koivunen et al., and Filtenborg et al. utilize population‐based cancer registries and aim to identify and study all cases in a defined population [4–6]. The principal drawback of such studies is a lack of detailed information, which can only be obtained by analysing patient records. Furthermore, as indicated by Filtenborg et al*.,* the case detection rate may vary, e.g., as reflected by an 8% discrepancy between the Danish Cancer Registry and the Danish Head and Neck Cancer Group for SNM [6]. Another disadvantage of registry-based studies is that inclusion of histological subtypes may not be uniform, and that treatment regimen may vary between contributing centres [2, 4–7]. Taken together, this highlights a need for population-based studies that include detailed crosschecking with patient records.

In this study involving consecutive patients in a population-based cohort from the Southern Swedish Health Care Region treated at one tertiary academic referral centre, we aimed to analyse characteristics and fate (survival) of SNM based on clinical information (not registry data). Accordingly, overall survival (OS), disease-free survival (DFS), and disease-specific survival (DSS) were analysed in relation to age, gender, site, stage, histopathology, and treatment.

## Methods

### Study design and patients

The study was of a retrospective design and involved consecutive patients with SNM from the population-based 1.9 million cohort of inhabitants of the Southern Swedish Health Care Region. These patients were either diagnosed at Skåne University Hospital, Lund, or referred to us from the five sub-regional hospitals, which reflects that treatment of all patients with SNM in the region is centralized to Skåne University Hospital. All patients from the Southern Swedish Health Care Region diagnosed with a primary SNM between January 1, 2001 and December 31, 2019 were identified using ICD-10 codes C300 to C318 and crosschecked against the regional multidisciplinary tumour (MDT) board registry. Ethical approval was granted by the regional Ethics Review Board (2018/745). Patient records were scrutinized and data on age, gender, subsite, stage, and histopathology were collected as were diagnosis date and treatment. Assessment of histological margin status is difficult in SNM, especially retrospectively, and was not included in the analysis. Demographics and survival outcome were analysed. Patients with earlier sinonasal cancer were excluded from the study.

### Staging

If applicable, patients were restaged according to the 8th Edition of the Union for International Cancer Control (UICC) TNM Classification of Malignant Tumors. Clinical information, including radiological findings, were obtained and used for restaging. Data were retrieved from the pathology reports and the following histopathologies were included: squamous cell carcinoma (SCC), sinonasal mucosal melanoma (SNMM), adenocarcinoma, salivary carcinoma, esthesioneuroblastoma (ENB), sinonasal undifferentiated carcinoma (SNUC), sinonasal neuroendocrine carcinoma (SNEC), and NUT carcinoma.

### Treatment and follow-up

Data on treatment intent as well as administered treatment were collected with date and type of surgical resection as well as dates and doses of administered RT with or without concomitant chemotherapy (RT ± CT). The therapy was categorized into the following groups: definitive RT ± CT, surgery alone, surgery with neoadjuvant or adjuvant RT ± CT. Treatment was also categorized into monotherapy or multimodality regimes that included surgery with either neoadjuvant or adjuvant RT ± CT. RT was administered 5 days per week in daily doses of 1.8–3 Gray (Gy) per fraction once or twice daily. Follow-up was also recorded, including date and localization of any recurrence, date and cause of death, and date of last follow-up. Patients had clinical follow-up controls every 3rd–6th month either up to 5-year post-treatment, until December 31, 2020, or death.

### Analysis

Median follow-up time was calculated from end of treatment to last follow-up or death. Disease free survival (DFS) was calculated as the time from end of primary treatment to the date of an event. Disease-specific survival (DSS) and overall survival (OS) were measured from date of diagnosis to date of death. Surviving patients still recurrence-free and/or alive at their last follow-up were censored on that date. Standard Kaplan–Meier estimates of DFS, DSS, and OS distributions were computed using SPSS 25.0. Differences between survival curves were assessed by the log-rank test. Analysis of prognostic factors was performed using Cox proportional hazard analyses.

## Results

Two-hundred and forty-three SNM patients were identified. Three patients under the age of 18 (1 NUT-sarcoma and 2 rhabdomyosarcoma) were excluded from the analysis, as were 9 patients with other types of sarcoma and 5 with hemangiopericytoma. Accordingly, 226 patients were analysed. Demographics, site, stage, histopathology, and treatment data are presented in Table 1. SCC was the most common subtype (53.1%), followed by SNMM (12.8%), adenocarcinoma (10.2%), salivary carcinoma (8.8%), ENB (6.6%), SNUC (5.8%), SNEC (2.2%), and NUT (0.4%). Eighty percent completed treatment with curative intent. The treatment consisted of multimodality regimes including surgery with neoadjuvant (29%) or adjuvant (37%) RT ± CT, as well as monotherapy regimes with surgery (22%), definitive RT + CT (7%), or RT (5%). Follow-up was 100%. The median follow-up time was 106 (95% CI 94–119) months.

**Table 1:** Demographics, tumor characteristics, treatment intention, and outcome by histologic subtype for 226 patients with sinonasal malignancies

	All	SCC	SNMM	Adeno	Salivary ^a^	ENB	SNUC	SNEC	NUT
	(*n* = 226)	(*n* = 120)	(*n* = 29)	(*n* = 23)	(*n* = 20)	(*n* = 15)	(*n* = 13)	(*n* = 5)	(*n* = 1)
Age									
Mean/median	66.0/67	67.5/68	68.3/69	61.1/65	60.6/60	62.7/67	71.7/69	58.8/54	40
Range	27–97	40–91	51–84	31–83	30–93	41–78	52–97	27–90	40
Sex (male) (%)	57	68	45	52	40	53	39	40	0
Stage^b^ (%)									
I	16	24	NA	13	10	13		20	
II	12	9	NA	30	30	13			
III	11	14	14	9	5				
IVA	31	28	69	26	20	27	23		
IVB	26	22	10	22	30	47	69	60	
IVC	4	3	7		5		8	20	100
Primary site (%)									
Nasal cavity	58	54	72	57	65	73	31	60	
Maxillary	22	29	10	22	20		8		100
Ethmoid	3	3	3	13					
Frontal	0	1							
Sphenoid	4	4	3		5		8		
Overlapping^c^	14	9	10	9	10	27	54	40	
Curative treatment intention (%)									
Initiated	84	83	93	96	70	100	69	60	0
Completed	80	80	76	96	70	100	62	60	0
Survival (months)									
Mean OS ± SD	88.7 ± 5.6	77.7 ± 6.7	69.2 ± 10.9	120.6 ± 15.8	103.1 ± 19.2	135.1 ± 9.6	50.5 ± 12.6	29.4 ± 13.9	5
Median OS	76	51	69	117	88	147	42	9	5
95% CI	57–94	20–82	28–110	89–145	26–150	56–238	0–86	5–13	
5-year OS (%)	56.6	48.1	54.9	77.3	70.0	100	44.0	20.0	0
10-year OS (%)	35.1	34.6	29.1	48.9	46.7	79.5	0	0	

*SCC* squamous cell carcinoma, *SNMM* sinonasal mucosal melanoma, *Adeno* adenocarcinoma, *Salivary* salivary carcinoma, *ENB* esthesioneuroblastoma, *SNUC* sinonasal undifferentiated carcinoma, *SNEC* sinonasal neuroendocrine carcinoma, *NUT* NUT carcinoma

^a^The histology for the 20 patients diagnosed with salivary carcinoma was adenoid cystic cancer (*n* = 13), salivary duct carcinoma (*n* = 2), epithelial–myoepithelial carcinoma (*n* = 2), acinic cell cancer (*n* = 1), mucoepidermoid cancer (*n* = 1), and cancer ex pleomorphic adenoma (*n* = 1). The 5-year OS rate for patients with adenoid cystic cancer was 76.9% (median OS was 86 months)

^b^Stage according to UICC TNM 8th edition

^c^Overlapping lesion of accessory sinuses (C31.8)

### Overall survival

**Fig. 1:**
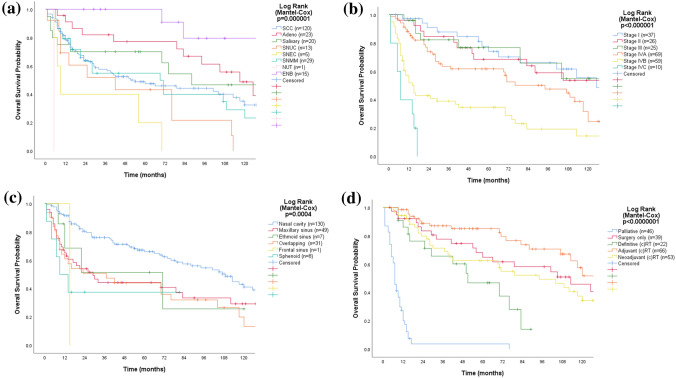
Kaplan–Meier curves displaying overall survival by histopathology (**a**), stage (**b**), site (**c**), and treatment (**d**) for 226 patients with SNM. *SCC* squamous cell carcinoma, *Adeno* adenocarcinoma, *Salivary* salivary carcinoma, *SNUC* sinonasal undifferentiated carcinoma, *SNEC* sinonasal neuroendocrine carcinoma, *SNMM* sinonasal mucosal melanoma, *ENB* esthesioneuroblastoma, *NUT* NUT-carcinoma, *RT ± CT* radiotherapy with or without concomitant chemotherapy

Median OS for all patients was 76 (95% CI 57–94) months with 5- and 10-year OS rates of 57% and 35%, respectively. Patients with ENB and adenocarcinoma had best prognosis, salivary carcinoma intermediate prognosis, whereas other histopathologies had poorer prognosis (Fig. 1a). The median OS by stage was 125 months for stage I (*n* = 37), 146 for stage II (*n* = 26), 140 for stage III (*n* = 25), 94 for stage IVA (*n* = 69), 15 for stage IVB (*n* = 59), and 7 for stage IV C (*n* = 10) (Fig. 1b). Patients with tumors located in the nasal cavity had significantly better survival compared to other subsites (Fig. 1c). The median OS by subsite was 109 months for nasal cavity, 71 for ethmoid sinus, 29 for maxillary sinus, 37 for overlapping lesions, 15 for frontal, and 9 for sphenoid sinus. As indicated in Fig. 1d, OS also varied significantly with treatment intent and modality (*p* < 0.001).

The results of the univariable and multivariable analyses for OS (risk for death) are listed in Table 2. Age, T and N-classification, site, stage, histopathology, and treatment were all significant risk factors (*p* < 0.001). Increasing age, stage IVA disease or higher, paranasal tumor localization, NUT histopathology, and treatment with palliative intention remained as significant independent negative prognostic factors in the multivariable analysis. There were no significant differences in survival between patients treated in the early (2001–2009) and the latter half (2010–2019) of the study period.

**Table 2:** Overall survival and risk for death in 226 patients with sinonasal malignancies

	*n*	Events	Univariable Cox			Multivariable Cox		
			*p* value	HR	(95% CI)	*p* value	HR	(95% CI)
All patients	226	126						
Age						0.002	1.03	1.01–1.04
0–65	99	43		1 (Ref.)		NA		
> 65	127	83	0.000	2.2	1.5–3.1			
Sex						NA		
Male	129	73		1 (Ref.)				
Female	97	53	0.404	0.9	0.6–1.2			
T classification						NA		
T1	38	15	0.000	1 (Ref.)				
T2	29	14	0.680	1.2	0.6–2.4			
T3	23	6	0.668	0.8	0.3–2.1			
T4a	68	37	0.051	1.8	1.0–3.3			
T4b	68	54	0.000	4.3	2.4–7.6			
N classification								
N0	204	110		1 (Ref.)		NA		
N +	22	16	0.000	2.6	1.6–4.5			
Stage								
I	37	14	0.000	1 (Ref.)		0.001	1 (Ref.)	
II	26	12	0.814	1.1	0.5–2.4	0.181	1.9	0.7–4.7
III	25	8	0.772	1.1	0.5–2.7	0.402	1.5	0.6–4.0
IVA	69	38	0.037	1.9	1.04–3.6	0.028	2.7	1.1–6.8
IVB	59	45	0.000	4.5	2.4–8.2	0.000	6.9	2.6–18.0
IVC	10	9	0.000	13.6	5.7–32.8	0.031	4.0	1.1–13.8
Tumor site								
Nasal cavity	128	61		1 (Ref.)			1 (Ref.)	
Paranasal	98	65	0.000	2.0	1.4–2.8	0.002	1.03	1.01–1.04
Histopathology								
SCC	120	65	0.000	1 (Ref.)		0.005	1 (Ref.)	
Adeno	23	11	0.052	0.5	0.3–1.0	0.080	0.5	0.3–1.1
Salivary	20	10	0.325	0.7	0.4–1.4	0.263	0.7	0.3–1.4
SNUC	13	11	0.084	1.8	0.9–3.3	0.012	0.4	0.2–0.8
SNEC	5	5	0.019	3.0	1.2–7.5	0.187	1.9	0.7–4.9
SNMM	29	20	0.568	1.2	0.7–1.9	0.664	0.9	0.5–1.6
NUT	1	1	0.012	13.4	1.8–102	0.049	9.1	1.0–82.3
ENB	15	3	0.011	0.2	0.1–0.7	0.007	0.2	0.1–0.6
Treatment								
Surgery only	39	21	0.000	1 (Ref.)		0.000	1 (Ref.)	
Multimodality	119	48	0.421	0.8	0.5–1.4	0.145	0.6	0.3–1.2
Definitive RT ± CT	22	14	0.020	2.3	1.1–4–6	0.478	0.7	0.2–1.9
Palliative	46	43	0.000	17.2	9.3–31.7	0.001	5.4	2.0–14.3

Age (binary variable lit at 65 years) as well as T and N classification were pre-selected to be included in univariate analysis, whereas age as a continuous variable and stage were pre-selected to be included in multivariable model

*HR* hazard ratios from uni- and multivariable Cox regression, *NA* not applicable, *Ref.* reference, *RT ± CT* chemoradiotherapy that included chemotherapy in 7% of the cases, *SCC* squamous cell carcinoma, *SNMM* Sinonasal mucosal melanoma, *Adeno* adenocarcinoma, *Salivary* salivary carcinoma, *ENB* esthesioneuroblastoma, *SNUC* sinonasal undifferentiated carcinoma, *SNEC* sinonasal neuroendocrine carcinoma, *NUT* NUT carcinoma, *RT ± CT* radiotherapy with or without concomitant chemotherapy

### Treatment with curative intent

Treatment with curative intent was initiated in 84% of the 226 patients but could only be completed in 80%. Demographics, tumor characteristics, treatment, and outcome for the 180 patients who completed curative treatment are depicted in Table 3. Of these patients, 158 (88%) patients had surgery, 141 (78%) had RT, and 37 (21%) CT or combinations thereof. Monotherapy was administered to 44%, with only surgery to 22%, definitive chemoradiotherapy (RT + CT) to 7%, and RT to 5%. Multimodality treatment that included surgery and RT ± CT was administered to 66%. Furthermore, 52 patients (29%) received neoadjuvant oncological therapy, whereof 37 had neoadjuvant RT and 15 had neoadjuvant RT + CT. Sixty-six patients (37%) received adjuvant oncological therapy, whereof 57 had adjuvant RT and 9 had RT + CT. No patient was subjected to neoadjuvant chemotherapy alone. Treatment varied significantly according to histology (Table 3) and stage (*p* < 0.001). Most stage I patients had only surgery (73%), most stage II and III surgery and adjuvant RT ± CT (50% and 56%, respectively), most stage IVA neoadjuvant (58%) or adjuvant (37%) RT ± CT in combination with surgery, and most stage IVB definitive RT ± CT (52%) or surgery with adjuvant RT ± CT (33%). The surgery was endoscopic in 19% and open transfacial in the remaining cases. It included medial maxillectomies in 44%, hemi maxillectomies in 25%, nose amputations in 8%, craniofacial resections in 6%, orbital eviscerations in 6%, and reconstruction with microvascular free flaps in 4%.

**Table 3:** Demographics, tumor characteristics, and outcome by histologic subtype for 180 patients treated with curative intent

	All	SCC		SNMM	Adeno	Salivary^a^	ENB	SNUC	SNEC
	(*n* = 180)	(*n* = 96)		(*n* = 22)	(*n* = 22)	(*n* = 14)	(*n* = 15)	(*n* = 8)	(*n* = 3)
Age									
Mean/median	63.8/65	65.3/67		67.0/68	61.1/64	53.4/52	62.7/67	69.4/66	54/54
Range	27–91	40–91		51–83	31–83	30–86	41–78	62–81	27–81
Sex (male) (%)	55	66		41	50	36	53	25	33
Stage^b^ (%)									
I	21	30		NA	14	14	13		33
II	14	11		NA	43	43	13		
III	14	18		18	7	7			
IVA	33	28		77	29	29	27	12	
IVB	18	13		5	7	7	47	88	67
Primary tumor site (%)									
Nasal cavity	67	65		82	59	86	73	38	67
Paranasal	22	29		9	32	7		12	
Overlapping^c^	11	6		9	9	7	27	50	33
Treatment (%)									
Surgery only	22	28		14	23	21			33
Definitive RT ± CT^d^	12	14					7	75	67
Adjuvant	37	24		27	54	72	93	13	
Neoadjuvant	29	34		59	23	7		13	
Survival (months)									
Mean OS ± SD	107.7 ± 6.1	94.6 ± 7.4		81.2 ± 11.7	125.6 ± 15.7	144.9 ± 18.3	135.1 ± 9.7	69.4 ± 16.6	45.0 ± 18.4
Median OS	109	104		71	125	146	147	76	56
5-year OS (%)	70.2	59.6		65.9	80.9	100	100	58.3	66.7
10-year OS (%)	44.0	42.9		34.9	51.1	66.7	79.5	0	0
Mean DSS ± SD	141.1 ± 6.7	135.2 ± 6.7		86.5 ± 12.1	170.6 ± 13.0	155.8 ± 18.5	135.1 ± 9.6	69.6 ± 15.2	49.7 ± 2.53
Median DSS	NA	NA		107	NA	NA	147	76	70
5-year DSS (%)	82.8	80.8		70.0	89.8	100	100	58.3	66.7
10-year DSS (%)	65.9	75.4		37.1	83.4	66.7	79.5	38.9	0
Mean DFS ± SD	119.8 ± 7.2	118.5 ± 7.4		59.1 ± 12.9	138.1 ± 18.2	84.5 ± 16.6	108.2 ± 14.5	61.9 ± 15.3	20.6 ± 13.3
Median DFS	NA	NA		47	NA	84	116	58	9
5-year DFS (%)	63.4	71.2		35.3	68.9	50.3	83.9	38.9	33.3
10-year DFS (%)	54.0	71.2		18.8	68.9	37.7	42.0	38.9	33.3

*SCC* squamous cell carcinoma, *SNMM* sinonasal mucosal melanoma, *Adeno* adenocarcinoma, *Salivary* salivary carcinoma, *ENB* esthesioneuroblastoma, *SNUC* sinonasal undifferentiated carcinoma, *SNEC* sinonasal neuroendocrine carcinoma, *RT ± CT* radiotherapy with or without concomitant chemotherapy, *OS* overall survival, *DSS* disease-specific survival, *DFS* disease free survival

^a^Salivary carcinoma including adenoid cystic carcinoma and mucoepidermoid carcinoma

^b^Stage according to UICC TNM 8th edition

^c^Overlapping lesion of accessory sinuses (C31.8)

^d^Definitive RT ± CT included chemotherapy in 7% of the cases

### Failures and disease-free survival

**Fig. 2:**
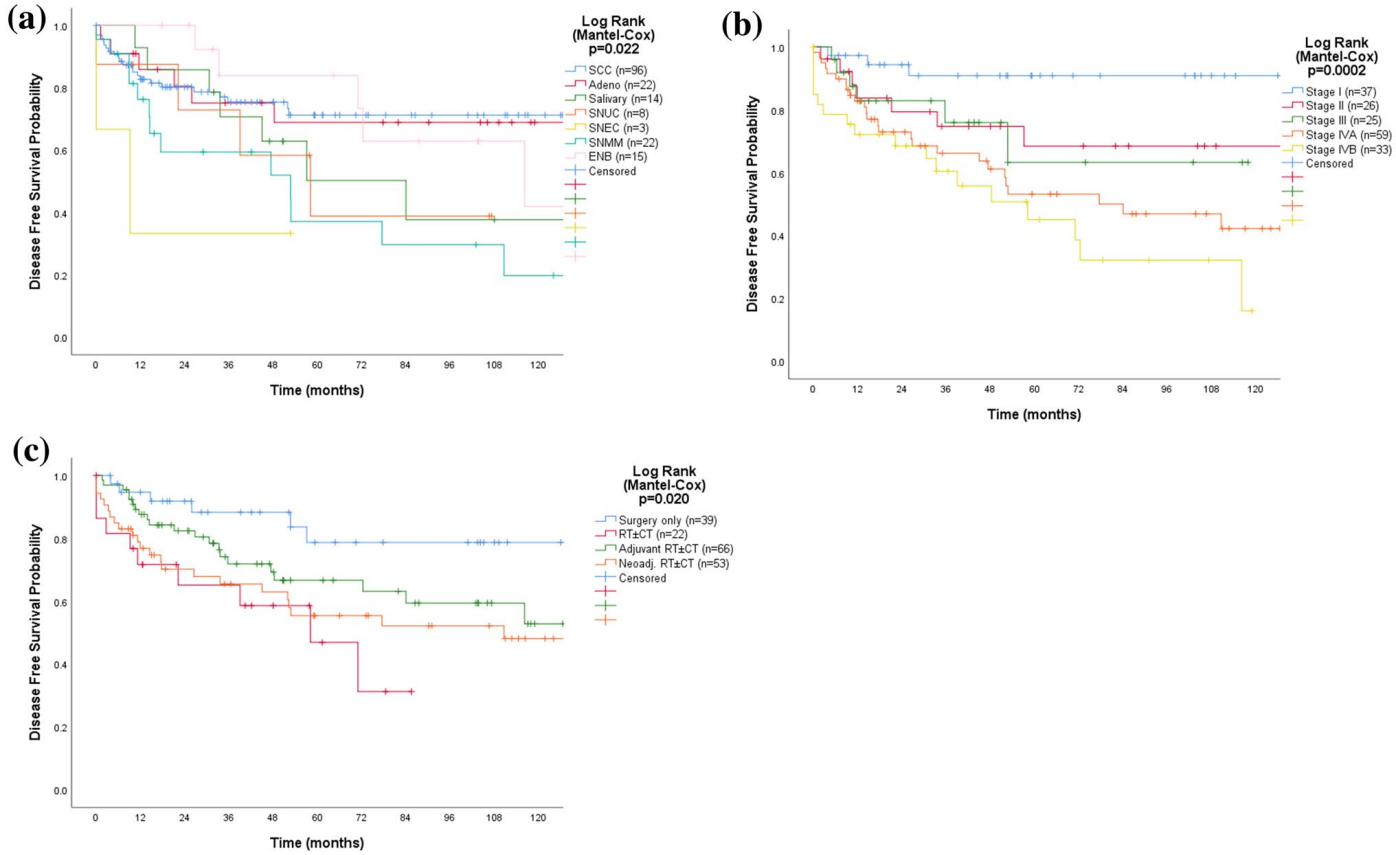
Kaplan–Meier curves displaying disease free survival by histopathology (**a**), stage (**b**), and treatment (**c**) for 180 patients with SNM treated with curative intent. *SCC* squamous cell carcinoma, *Adeno* adenocarcinoma, *Salivary* salivary carcinoma, *SNUC* sinonasal undifferentiated carcinoma, *SNEC* sinonasal neuroendocrine carcinoma, *SNMM* sinonasal mucosal melanoma, *ENB* esthesioneuroblastoma, *RT ± CT* radiotherapy with or without concomitant chemotherapy

During follow-up, 31% (*n* = 55) of the 180 patients treated with curative intent suffered from recurrences (*i.e.,* 17 local, 15 regional, 17 distant, 2 loco-regional, 2 regio-distant, and 2 all sites). Six patients were never in remission: one stage IVA SCC and 5 stage IVB (2 SCC, 1 SNMM, 1 SNUC, 1 SNEC). The DFS varied significantly according to histopathology (*p* = 0.022), stage (*p* = 0.0002), and treatment modality (*p* = 0.020), but not according to primary site (*p* = 0.056) (Fig. 2). SCC, adenocarcinoma, and ENB had the longest DFS, whereas patients with SNMM, SNEC, and SNUC had the shortest (Table 3). Patients who only had surgery (*n* = 39) had the longest DFS (mean 119.4 ± 9.3 months), whereas the 22 patients who received definitive RT ± CT (whereof 13 in combination with concomitant CT) had mean DFS of 49.8 ± 8.0 months. Patients who received multimodality treatment with RT ± CT in a neoadjuvant setting (*n* = 53) had a mean DFS of 107.8 ± 12.7 months and 105.2 ± 9.3 months in the adjuvant setting (*n* = 66).

T-classification (*p* = 0.002), regional metastases (*p* = 0.005), stage (*p* = 0.002) histopathology (*p* = 0.020), and treatment (*p* = 0.033), but not age or gender had significant impact on risk for recurrences or never being in remission (univariable analysis). According to multivariable regression analysis, stage (*p* = 0.001) and histopathology (*p* = 0.030) remained as significant independent factors for recurrence: stage III (HR 4.6 [95% CI 1.1–18.4]), stage IVA (HR 5.9 [95% CI 1.7–20.8]), stage IVB (HR 12.8 [95% CI 3.6–45.2]), SNEC (HR 5.0 [95% CI 1.1–23.1]), and melanoma histopathology (HR 2.2 [95% CI 1.1–4.5]).

### Disease-specific survival

**Fig. 3:**
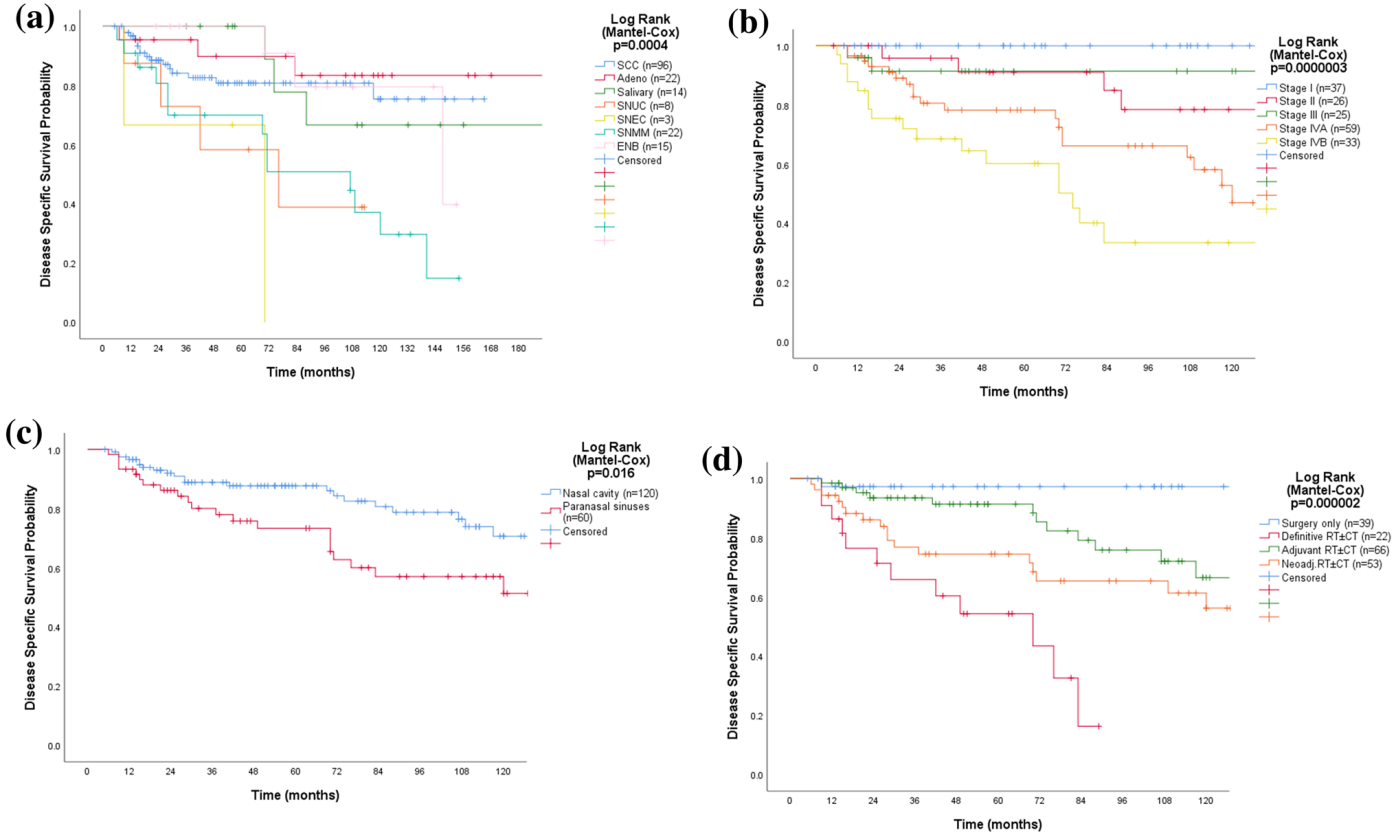
Kaplan–Meier curves displaying disease-specific survival by histopathology (**a**), stage (**b**), site (**c**), and given treatment (**d**) for 180 patients with SNM treated with curative intent. *SCC* squamous cell carcinoma, *Adeno* adenocarcinoma, *Salivary* salivary carcinoma, *SNUC* sinonasal undifferentiated carcinoma, *SNEC* sinonasal neuroendocrine carcinoma, *SNMM* sinonasal mucosal melanoma, *ENB* esthesioneuroblastoma, *RT ± CT* radiotherapy with or without concomitant chemotherapy

During follow-up, 44 of the 180 patients treated with curative intent succumbed to their disease and 38 died of intercurrent disease. The DSS varied significantly according to stage (*p* = 0.0000003), histopathology (*p* = 0.0004), treatment modality (*p* = 0.000002), and primary site (*p* = 0.016) (Fig. 3). The 5- and 10-year OS and DSS rates by histopathology are presented in Table 3. Patients with adenocarcinoma and salivary carcinoma had the longest DSS, whereas patients with SNUC and SNEC had the shortest. Patients who only had surgery had the longest DSS (mean 150.5 ± 5.0 months), whereas patients who received definitive RT ± CT had a mean DSS time of 54.9 ± 6.9 months. Patients who received multimodality treatment with RT ± CT in the neoadjuvant setting had a mean DSS of 131.8 ± 12.1 months, whereas it was 148.9 ± 10.6 months in the adjuvant setting (*n* = 66). Increasing age (1-year increments) (HR 1.04 [95% CI 1.02–1.06]), stage IVB disease (HR 4.6 [95% CI 2.1–10.0]), melanoma histopathology (HR 4.7 [95% CI 2.0–10.9]), and treatment with definitive RT ± CT (*p* = 0.001, HR 17.3 [95% CI 3.3–90.9]) remained as significant independent prognostic factors for disease-specific mortality (DSM) according to multivariable regression analysis (*p* ≤ 0.001).

### Stage IVB disease

**Fig. 4:**
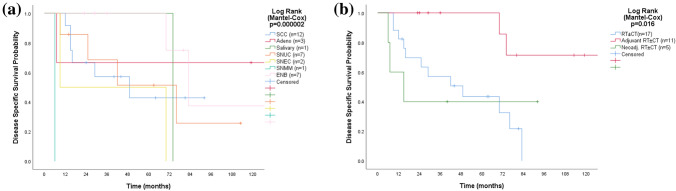
Kaplan–Meier curves displaying disease-specific survival by histopathology (**a**) and treatment modality (**b**) for 33 patients with stage IVB SNM treated with curative intent. *SCC* squamous cell carcinoma, *Adeno* adenocarcinoma, *Salivary* salivary carcinoma, *SNUC* sinonasal undifferentiated carcinoma, *SNEC* sinonasal neuroendocrine carcinoma, *SNMM* sinonasal mucosal melanoma, *ENB* esthesioneuroblastoma, *RT ± CT* radiotherapy with or without concomitant chemotherapy

Fifty-nine patients in the total cohort were diagnosed with stage IVB disease. During follow-up, 42 succumbed to their malignancy and 3 died of intercurrent disease. The median OS was 15 months. OS varied significantly by histopathology (*p* = 0.003) and was longest for ENB (median 83 months) while shortest for salivary carcinoma (median 4 months), SNMM (median 7 months), and SNEC (median 9 months). Treatment with curative intent was administered to 56% (*n* = 33) despite that 20 of those patients had intracranial, 12 sphenoidal, and/or 7 nasopharyngeal involvements. Six patients had tumors involving the orbital apex and 5 the retro-maxillary space. Treatment varied significantly according to histopathology (*p* = 0.005). All 33 patients subjected to curative treatment received RT. Seventeen had definitive RT ± CT (8 SCC, 6 SNUC, 2 SNEC) and 16 had surgery after neoadjuvant RT ± CT (3 SCC, 1 Adeno, 1 SCC) or before adjuvant RT ± CT (6 ENB, 2 Adeno, 1 SCC, 1 salivary, 1 SNUC). The RT ± CT included concomitant CT in 15 cases*, i.e*., for 65% in the definitive RT ± CT group, 27% in the adjuvant, and 20% in the neoadjuvant group. The surgery was endoscopic in 9 cases (mostly ENB) and open transfacial in 24. Six patients were never in remission (2 after neoadjuvant treatment and surgery and 4 after definitive RT ± CT. During follow-up, 12 additional patients succumbed to their malignancy. The DSS varied significantly according to histopathology (*p* = 0.00002) and was longest for adenocarcinoma and ENB while shortest for SNMM and SNEC (Fig. 4a). Survival also varied significantly according to treatment modality (*p* = 0.016). The 5-year DSS was 100% after adjuvant RT ± CT, whereas it was 40% after neoadjuvant RT ± CT plus surgery and 43% after definitive RT ± CT (Fig. 4b). Having a tumor deemed as not resectable increased risk for DSM (*p* = 0.006, HR 6.1 [95% CI 1.7–22.1]). Having surgery and adjuvant RT ± CT reduced the risk for DSM *cf.* definitive and neoadjuvant RT ± CT (*p* = 0.015, HR 0.2 [95% CI 0.0–0.7]).

### Patients treated with palliative intent

Treatment with palliative intent was administered to 20% of the cohort (*n* = 46). The median age was 76 (range 40–97) years and 35% were women. These patients were significantly older than the patients who completed treatment with curative intent (*p* < 0.001). The histopathology was SCC in 52%, SNMM in 15%, salivary carcinoma in 13%, SNUC in 11%, SNEC in 4%, adenocarcinoma in 2%, and NUT-sarcoma in 4%. All presented with stage IV disease (stage IVA 22%, IVB 56%, and IVC 22%). Best supportive care was administered to all patients, whereof 52% also received palliative RT, 20% palliative RT + CT, and 6% palliative surgery. The mean survival was 10.3 ± 2.4 months. The 1- and 2-year OS rates were 28.3% and 3.9%, respectively.

## Discussion

This study describes characteristics and fate for 226 consecutive patients with de novo SNM, including SNMM and ENB, who were treated at a single tertiary academic referral centre. The lead finding is a seemingly good survival outcome, especially for patients with SNMM, adenocarcinoma, and SNUC. Together with published literature on the matter, our results may provide a background for future RCTs. The strength of our report reflects that it involves a population-based cohort and that curated data have been obtained directly from patient records.

A principal feature of our findings is a seemingly longer survival for the whole cohort (including patients treated with curative and palliative intent) compared to other studies. The 5-year OS rate of 57% is numerically better than corresponding rates reported by Thorup et al. (47%), Koivunen et al. (38%), Filtenborg et al. (46%), and Gore et al. (46%) [4–7]. To the best of our understanding, these discrepancies are probably not explained by factors, such as age, site, or even stage. In fact, a greater percentage of our patients presented with stage IV disease (61%), *cf*. Thorup (48%), Koivunen (52%), and Filtenborg (58%). Similarly, the apparently better survival in our study *cf*. Gore et al. may not be explained by less advanced tumors, as regional and distant metastases, respectively, were present in 9.5% and 4.1% in our study *cf*. in 4.4% and 1.5% in the U.S. cohort [7]. Moreover, the 5-year DSS rate of 83% for patients treated with curative intent in our cohort is higher than in the two registry-based studies from Denmark reported by Thorup et al. (57%) and Filtenborg et al. (56%) [4, 6]. However, we fully concede that cohort heterogeneity makes it difficult to reach definitive conclusions regarding survival differences between our study and the Nordic [4–6] and U.S. [2, 7] registry-based reports, i.e., differences in distributions of histopathologies, nasal/paranasal localizations, and tumour stages as well as differences in distributions between treatment with palliative and curative intent.

It is essential to consider the histopathology of SNM in relation to specific outcome measures, because it is an important prognostic factor [8]. Our findings corroborate this, because histopathology influenced DFS, DSS, and OS. Patients with adenocarcinoma and ENB had the best prognosis, while SCC, SNMM, salivary carcinoma, and SNUC had poorer, and SNEC and NUT had the worst prognosis. In 2017, Robin et al*.* presented survival for SNM per histopathology based on 11,160 patients from of the *National Cancer Database*, representing > 1,500 community and academic cancer centres and approximately 70% of all cases in the U.S. [2]. SCC was most frequent (54%) followed by SNMM (10%), adenocarcinoma (7%), adenoid cystic cancer (7%), SNUC (4%), and ENB (10%). These percentages are similar to what we observed, and heterogeneity in histopathology may not explain the differences in outcomes between the reports. Robin et al*.* reported no survival rates but instead median OS times: 52.8 months for SCC (*cf*. 54 months in our cohort), 22.4 months for SNMM (*cf*. 69 months in our cohort), 98.6 months for adenocarcinoma (*cf*. 117 months in our cohort), 86.1 months for adenoid cystic cancer (*cf*. 86 months in our cohort), and 33.4 months for SNUC (*cf*. 42 months in our cohort). In the study by Robin et al., median OS was not reached for ENB, whereas it was 147 months in our cohort.

Survival for patients with SNM of specific histopathologies in the present study seems to surpass what was reported by Robin et al. [2]. The most pronounced difference concerns SNMM. As indicated above, we observed 46 months longer median survival time for SNMM patients than presented by Robin et al. [2]. Moreover, the observed 5-year OS rate of 55% for all SNMM in the present study agrees with and exceeds the OS previously reported by our group (that included a part of the present study population) [9]. The 55% OS rate also compares favourably to other studies that generally have indicated 5-year OS rates of less than 40% for SNMM [7, 10–14]. As discussed in our earlier publication [9], the differences might reflect our frequent utilization of neoadjuvant hyper-fractionated RT with concomitant chemotherapy or adjuvant RT following surgery for stage IVA SNMM. In agreement, recent systematic meta-analyses indicates that adjuvant RT prolongs survival for patients with SNMM [15, 16], while others suggest adjuvant RT does not prolong survival yet increases local control [17]. From the limited number of 17 patients with stage IVA SNMM in this study, the only significant difference that emerged in the context of neoadjuvant/adjuvant measures was that patients subjected to RT + CT (*n* = 12) had a longer OS (mean 81.9 ± 13.2, median 71 months) than those who only received RT (*n* = 5) in addition to the surgery (mean 22.5 ± 2.9, median 21 months) (*p* = 0.046). According to multivariable regression analysis, treatment with RT ± CT *vs*. RT remained as a significant independent prognostic factor for survival reducing the risk of death for stage IVA patients (HR 0.16 [95% CI 0.029–0.924]) after adjusting for age, and neoadjuvant or adjuvant treatment regimens (*p* = 0.040). Taken together, our observations suggest the possibility that patients with advanced SNMM may benefit from concomitant RT + CT before surgery, but of course RCTs focusing on adjuvant therapies are warranted.

Despite recent therapeutic advances, the prognosis for SNUC remains poor [18]. One of our observations was a seemingly good outcome for patients with SNUC with reservation for the small subset of eight patients. The median OS was 42 months compared to 33 months reported by Robin et al. [2]. Multimodal therapy is a well-established approach for treating SNUC [19]. For example, in a cohort of 95 patients with SNUC who received induction chemotherapy ahead of definitive therapy, 5-year DSS was 59% [19]. The 5-year DSS of 58% was similar in our cohort of SNUC-patients subjected to treatment with curative intent. However, only two patients received concomitant RT + CT and the other six RT (combined with surgery in two cases). This outcome corroborates findings by Lehrich et al., in a study involving 440 patients, indicating that induction chemotherapy may not provide survival benefits in SNUC patients [20]. As for many other subsets of SNM, further studies are warranted also for SNUC. Arguably, such may involve selecting patients for definitive treatment based on response to chemotherapy (a.k.a. chemoselection), as suggested by Amit et al. [19], or based on overall immune phenotype, as suggested for other malignancies [21]**.** Another conspicuous finding in our study was the long survival for patients with adenocarcinoma (*n* = 23). The median OS was 18 months longer than presented by Robin et al. [2]. We report DSS-rates of 89% and 83% as well as OS-rates of 77% and 49% after 5 and 10 years, respectively. These rates can be compared to a Norwegian cohort (*n* = 20) with DSS-rates of 73% and 58% and OS-rates of 68% and 54% after 5 and 10 years, respectively [22]. Again, RCTs are warranted stratified per histology, site and stage.

Despite that ENB was described already in 1924, and given the rarity of the malignancy (3–6% of all SNM), no consensus has been reached regarding staging and optimal treatment strategy [23]. The most widely accepted, yet unofficial, classification for ENB was proposed by Kadish et al. in 1976 [24]. However, other proposed staging systems include the conventional TNM staging by the UICC as well as a modified version of the TNM classification proposed by Dulguerov in 1992 [23, 25]. We chose to use the UICC TNM system to better be able to compare the outcome for ENB with other histopathologies. We observed an apparent high survival for ENB. Both the DSS and OS rates were 100% at 5 years and 79.5% at 10 years. In comparison, Jethanamest et al. presented much lower OS rates (62.1% at 5 years and 45.6% at 10 years) in a cohort of 311 ENB patients despite that the mean age was lower than in our cohort (mean age 54 *cf.* 63 years) [26]. Moreover, in another cohort of 26 ENB patients, 74% were alive at 5 years and 60% at 10 years [25]. The apparent better outcome in our cohort may be a coincidence due to comparatively few patients. Another explanation may be that all ENB patients, except one who declined surgery, received multimodality therapy with surgery and adjuvant RT ± CT.

Stage IVB disease infers a dismal prognosis and stage IVB patients are often subjected to aggressive treatment. Therefore, conscientious considerations of treatment sequelae and survival probability are essential. In our cohort, 26% were diagnosed with stage IVB disease. More than half of these patients were treated with curative intent that included RT in all cases and surgery in almost half. A tumor not considered resectable significantly increased the risk for DSM. Accordingly, this meant a treatment regimen that did not include surgery. A couple considerations may be made in this context. First, there are histopathological subsets of stage IVB disease where surgery seems to be futile, e.g., SNMM. Second, there may be other subsets, e.g., ENB, salivary carcinoma, and adenocarcinoma, where a gain by surgery if combined with adjuvant therapy may well offset any morbidity. Indeed, the 5-year DSS was 100% for stage IVB patients who received adjuvant RT ± CT *cf*. 43% after definitive RT ± CT and 40% after neoadjuvant RT ± CT, suggesting a benefit of multimodality treatment regime of surgery with adjuvant RT ± CT. This was confirmed through the multivariable regression analysis, implying that adjuvant RT ± CT significantly reduced the risk for DSM (*cf*. definitive and neoadjuvant RT ± CT). However, except for resectabilty, no other factors, such as age, gender, site, stage, histopathology, or chemotherapy affected risk for DSM. In this study, 19% of the surgical procedures were endoscopic and the remainder open. In part this may reflect a development over time, with open surgery being standard and endoscopic surgery becoming more common, but sometimes also a deliberate strategy to avoid any piecemeal resection of the tumour. However, the numbers in this study are too low to allow for a comparison between endoscopic and open approaches stratified per histology, site, and stage. To the best of our understanding, there are no RCTs comparing aspects of survival between two surgical approaches for SNM, but such studies are warranted.

In conclusion, our results indicate a seemingly good outcome for SNM patients compared to previous reports, particularly for SNMM, adenocarcinoma, and SNUC. The strength is that the study is population-based and involves consecutive patients assessed and treated at a single tertiary academic referral centre. On the other hand, a weakness is that the material is not stratified for histological margin status. However, such an analysis is very difficult to perform retrospectively, since the question of microscopic radicality and tumour margins frequently involves a close discussion between the pathologist and the surgeon even for *en* *bloc* resections. Nevertheless, our observations emphasize the importance of histopathology as a risk factor for SNM and indicate a need for multi-centre RCTs stratified per histopathology and possibly involving multimodality strategies.
