# Correction to: Outcome for sinonasal malignancies: a population-based survey

**DOI:** 10.1007/s00405-022-07329-3

**Published:** 2022-03-12

**Authors:** Anna Hafström, Johanna Sjövall, Simon S. Persson, Johan S. Nilsson, Christer Svensson, Eva Brun, Lennart Greiff

**Affiliations:** 1 Department of ORL, Head & Neck Surgery, Skåne University Hospital, 221 85 Lund, Sweden; 2 Department of Clinical Sciences, Lund University, Lund, Sweden; 3 Department of Oncology, Skane University Hospital, Lund, Sweden

## Correction to: European Archives of Oto-Rhino-Laryngology (2021) 10.1007/s00405-021-07057-0

In the original publication of the article, the following author name “Johan S. Nilsson” was missing and included in this correction as fourth author.

The respective affiliation should read as below,

1. Department of ORL, Head & Neck Surgery, Skåne University Hospital, 221 85 Lund, Sweden

2. Department of Clinical Sciences, Lund University, Lund, Sweden

The original article has been corrected.

